# Differentials and income-related inequalities in maternal depression during the first two years after childbirth: birth cohort studies from Brazil and the UK

**DOI:** 10.1186/1745-0179-5-12

**Published:** 2009-06-05

**Authors:** Alicia Matijasevich, Jean Golding, George Davey Smith, Iná S Santos, Aluísio JD Barros, Cesar G Victora

**Affiliations:** 1 Post Graduate Programme in Epidemiology, Federal University of Pelotas, RS, Brazil; 2 Department of Community Based Medicine, University of Bristol, UK; 3 Department of Social Medicine, University of Bristol, UK

## Abstract

**Background:**

Depression is a prevalent health problem among women during the childbearing years. To obtain a more accurate global picture of maternal postnatal depression, studies that explore maternal depression with comparable measurements are needed. The aims of the study are: (1) to compare the prevalence of maternal depression in the first and second year postpartum between a UK and Brazilian birth cohort study; (2) to explore the extent to which variations in the rates were explained by maternal and infant characteristics, and (3) to investigate income-related inequalities in maternal depression after childbirth in both settings.

**Methods:**

Population-based birth cohort studies were carried out in Avon, UK in 1991 (ALSPAC) and in the city of Pelotas, Brazil in 2004, where 13 798 and 4109 women were analysed, respectively. Self-completion questionnaires were used in the ALSPAC study while questionnaires completed by interviewers were used in the Pelotas cohort study. Three repeated measures of maternal depression were obtained using the Edinburgh Postnatal Depression Scale in the first and second year after delivery in each cohort. Unadjusted and adjusted analyses were carried out. The Relative index of Inequality was used for the analysis of income-relate inequalities so that results were comparable between cohorts.

**Results:**

At both the second and third time assessments, the likelihood of being depressed was higher among women from the Pelotas cohort study. These differences were not completely explained by differences in maternal and infant characteristics. Income-related inequalities in maternal depression after childbirth were high and of similar magnitude in both cohort studies at the three time assessments.

**Conclusion:**

The burden of maternal depression after childbirth varies between and within populations. Strategies to reduce income-related inequalities in maternal depression should be targeted to low-income women in both developed and developing countries.

## Background

Depression is a prevalent health problem among women during the childbearing years. Pregnancy and postpartum periods are windows of the highest vulnerability to depression [1]. Postpartum depression has been associated with serious negative effects on familial relationships [2]. It can also impair infant care and increase the risk of infant cognitive and emotional delay, medical problems and hospitalizations [3,4].

Some risk factors that have been associated with maternal depression after childbirth are: poor social support, marital conflict, adverse life events, low maternal education, poverty, belonging to minority racial or ethnic groups and young maternal age [5,6]. The strongest predictors of postpartum depression are depressive symptoms during pregnancy or a history of depression before pregnancy [5].

Prevalence rates of maternal depression after childbirth vary both within and across countries, ranging from as low as 4% [7] to as high as 74% [8]. However, the scarcity of large surveys from developing countries, the variability of instruments used for the diagnosis with varying cut-off scores, different sampling methodologies and the timescale of the studies, among other difficulties, have hindered making valid comparisons [9].

The present study aimed to compare the prevalence of maternal depression in the first and second year postpartum between two population-based birth cohort studies carried out in the county of Avon, UK and in the city of Pelotas, Brazil, where similar methodology was used to ascertain maternal depression. We explore the extent to which variations in rates of maternal depression after childbirth were explained by characteristics of the mothers and their offspring. Additionally family income inequalities in maternal depression in the first and second year postpartum were investigated in both settings.

## Methods

### Study population

In the Avon health authority area in south-west England in 1991–1992 and in the city of Pelotas, Brazil, in 2004, population-based cohort studies were carried out. The populations of these studies belong to two contrasting cultures with different levels of development. While the UK is a high income-country, Brazil is not only a middle-income country, but also one of world's most socially unequal countries [10].

ALSPAC (the Avon Longitudinal Study of Parents and Children) was specifically designed to determine ways in which the individual's genotype combines with environmental pressures to influence health and development. A detailed description of the methodology of this study is given elsewhere[11,12]. The ALSPAC study started during pregnancy and aimed to enrol all women who were resident in the three Bristol-based health districts of the county of Avon (population 940,000) and who had an expected date of delivery between April 1, 1991 and December 31, 1992. Approximately 85% of the eligible mothers in the study area took part. Information was obtained both from self-completion questionnaires and from clinical records. The present paper uses the information collected from six self-completion questionnaires. Two were sent to the pregnant women during pregnancy (at 18 and 32 weeks of gestation), and four subsequently at eight weeks and eight, 21 and 33 months postnatally.

The city of Pelotas, located in Southern Brazil, has a population of about 330,000, and more than 99% of all deliveries take place in hospitals. During the entire year of 2004, all births taking place in the city were recruited for a birth cohort study, excluding those mothers resident in other municipalities or in rural areas. A detailed description of the methodology is given elsewhere [13]. Births were identified by daily visits to the five maternity hospitals. Soon after delivery, mothers were interviewed using a pre-tested structured questionnaire. Detailed information was obtained about demographic, socioeconomic, behavioural and biological characteristics, reproductive history and health care utilization. The non-response rate at recruitment was below 1%. Children whose mothers lived in the urban area of Pelotas were visited at home at three, twelve and 24 months after birth. On each occasion, mothers were interviewed by trained field workers, using a standardized questionnaire, collecting information about mothers' and children's health.

Because in 2004 Pelotas cohort study women with perinatal or infant death were not followed-up, only women with live births who did not die in the first year of life were included in both cohort studies. The same variable definitions and comparable questions were used in the two studies.

### Data on outcome

In both cohort studies repeated measures of maternal depression were obtained using the Edinburgh Postnatal Depression Scale (EPDS) [14]. The EPDS was originally devised for the identification of postpartum depression disorders for use in clinical and research settings. EPDS is a self-administered, 10-item scale; each item has four possible responses from 0 to 3, with a minimum score of 0 and a maximum of 30. The scale expresses the intensity of depressive symptoms over the preceding seven days. The clinical and epidemiological value of the scale has been confirmed by several validation studies carried out in different countries, including, in Brazil, among a sample of mothers from the 2004 Pelotas birth cohort study [15]. In this study, the cut-off point validated for diagnosis of postpartum depression was ≥ 13, so the EPDS was dichotomized at <13 and ≥ 13 to produce a "non-depressed/depressed" classification. Sensitivities and specificities of 95% and 93% respectively have been achieved by identifying women with EPDS scores of ≥ 13 and comparing the result with clinical interview, using the DSM III criteria [15,16].

In the ALSPAC study, the results of the EPDS administered to mothers at eight weeks, eight months and 21 months postnatally were used. In the Pelotas cohort study, the EPDS was administered to a sample of mothers at the 3^rd ^month follow-up and to all mothers of children of the cohort at the twelve and 24-month follow-ups. The sample at the 3^rd ^month follow-up comprised all mothers whose babies reached an age of three months between 1^st ^January and 31 March 2005 (born, therefore, between 1^st ^October and 31 December 2004) including about one-fourth of all mothers of the cohort.

In the Pelotas study, in order to ensure the scale's adequacy, the ten questions were translated into Portuguese and back again into English. In contrast to the original self-administered format, questions were posed to mothers by a trained interviewer, as a single block and in the same order as in the original instrument, within the cohort's regular interviews. The decision to pose the questions to mothers verbally was related to the fact that an important proportion of mothers from the cohort had little schooling as well as being unfamiliar with self-administered data collection instruments. The administration of EPDS as an interview is accepted by the instrument's authors [14] and has been used previously [17].

### Data on determinants

The following factors were considered to be potential determinants for maternal depression after childbirth: mother's ethnic origin, family income, antenatal risk, maternal age at delivery (<20, 20–34 and 35+), marital status (women who were single, widowed, or divorced or who lived without a partner were classified as single mothers), parity (defined as the number of previous pregnancies resulting in a live birth or a late foetal death and categorised as 0, 1 and 2+), pre-pregnancy body mass index (categorized as < 18.5, 18.5-<25, 25-<30 and ≥ 30 kg/m^2^), smoking during pregnancy (smokers were those women who smoked at least one cigarette per day on an everyday basis in any trimester of pregnancy, categorized as yes or no), multiple birth (categorized as singleton or multiple) and neonatal problems (defined as admission to the special care baby unit or neonatal intensive care, categorized as yes or no).

Mothers' ethnic origin was self-reported in the ALSPAC study using the format asked in the 1991 United Kingdom Census. This categorises the person as White, Black/Caribbean, Black/African, Black/other, Indian, Pakistani, Bangladeshi, Chinese, Other Specified. In the Pelotas Cohort study, maternal skin colour was chosen as a proxy for ancestral background, because miscegenation in Brazil is highly prevalent [18] and it is not feasible to classify women into different ethnic groups in large-scale studies. Skin colour options given to the interviewers were white, black, and other. Because women in the Black and "other skin colour" or "other ethnic origin" categories had similar socio-demographic characteristics they were assembled into a single group in both cohort studies and the variable was categorized into White and Black/mixed ethnic origin.

Family income in the month prior to delivery was collected in the perinatal interview in the Pelotas cohort study. In the ALSPAC study family income per week was collected at 33 months after delivery. Because of the different currencies used in the ALSPAC and Pelotas birth cohort study and to allow comparison between studies, quintiles of income in each cohort were used.

The gestational risk score system of the 1970 British birth survey [19] adapted by Barros *et al*. [20] was used to classify women in both cohorts according to different levels of antenatal risk. The score included the following items: maternal age, parity, income, reproductive history (miscarriage, foetal death, infant death and low birthweight), history of diabetes, height <150 cm, marital status and smoking during pregnancy. Further explanation about the construction of the score can be found in a previous publication [21]. Those women with score ≤ 2 points were considered to be low risk, those with a score between 3 and 7 points were considered to be medium risk and those women above 7 points were considered to have high antenatal risk.

### Statistical analysis

We used χ^2 ^tests to compare the distribution of maternal characteristics between the ALSPAC and Pelotas birth cohort studies and to study the association between these characteristics and maternal depression after childbirth in each cohort at the three time assessments. The first assessment was at eight weeks and three months after delivery for the ALSPAC and the Pelotas birth cohort, respectively. The second assessment was at eight and twelve months and the third at 21 and 24 months postpartum for the ALSPAC and Pelotas birth cohort, respectively.

As some variables from the ALSPAC database had a high proportion of missing values – i.e. family income 37%, pre-pregnancy body mass index 18% and smoking during pregnancy 22%-multiple imputation was the method chosen for handling missing data problems. We used Stata^® ^release 9.2 to perform the multiple imputation [22]. The method for imputation and subsequent analysis of the filled-in data involved two steps. In the first step, all variables were imputed together allowing the missing values for each variable to be predicted from all of the other variables (using "ice" command). Five imputed complete datasets were created. Finally, to estimate the relationship between maternal depression after childbirth and cohort study, these data sets were analyzed by logistic regression using "mim" command to obtain the estimated odds ratios and 95% confidence intervals by combining the results of the imputed datasets. All the analyses were also done in the database with missing variables and the results were very similar as those using the database with multiple imputation (data available at request).

Adjusted analyses took into account the fact that the differences in maternal depression after childbirth between ALSPAC and Pelotas birth cohort studies could be mediated through maternal and infants' characteristics. To be included in the multivariate analyses, variables were to be associated with both maternal postnatal depression and cohort study, (P < 0.2) in at least one of the three time points studied. We evaluated the correlation matrix for any evidence of multicollinearity before finalizing the models.

To estimate inequalities in maternal depression after childbirth between ALSPAC and the Pelotas birth cohort studies, logistic regression analysis was used to calculate relative indices of inequality (RII) as a measure of maternal depression differential by family income [23]. To calculate the RII in both cohorts, an income position indicator was used as an independent variable in the logistic regression analysis. First the percentage of women in each category of family income was calculated. Then their relative position was obtained by deriving a score from 0 to 1, the lowest to highest family income, based on the mid-point of the proportion of the cohort in each category. For example, if 20% of the women in one of the cohorts were in the lowest income group and 20% in the next category, women in the lowest category would be assigned a value of 0.10 (0.20/2) and those in the second category a value of 0.30 [0.20 + 0.20/2], and so on for each category of family income. This income position indicator was then entered as an independent ordinal variable in the logistic regression analysis. The regression coefficient of the income position indicator and the standard error were subsequently used to calculate the odds ratio with 95% confidence intervals. This odds ratio is known as the RII. Results were interpreted as the comparison of the extremes – the lowest compared with the highest income position. For each time assessment we tested whether the RII in ALSPAC differed from that in the Pelotas cohort study, by including a cohort x income interaction term in the logistic regression model. All analyses were performed with Stata software version 9.2 (StataCorp LP, College Station, Texas, USA).

### Details of Ethics Approval

Ethical approval for the study was obtained from the ALSPAC Law and Ethics Committee and the Local Research Ethics Committees. The study protocol of 2004 Pelotas cohort study was approved by the Medical Ethics Committee of the Federal University of Pelotas, affiliated with the Brazilian Federal Medical Council.

## Results

The core ALSPAC study consisted of 14 541 pregnancies and after excluding stillbirths, abortions and infant deaths there remained 13 798 women for analysis. Response rates were 83.5%, 80.1% and 74.0% at eight weeks, eight months and 21 months after delivery. Missing information on the EPDS questionnaire was 1.2%, 1.3% and 1.6% at each time assessment, respectively.

The 2004 Pelotas birth cohort study consisted of 4287 births, and after excluding stillbirths and infant deaths there remained 4109 women for analysis. At the three-month follow-up after delivery, a sub-sample of 965 mothers was chosen for administration of the EPDS questionnaire. Response rate was 91.2% and all women completed each item of the EPDS questionnaire. Multiparae and women with multiple birth were more frequent among the sample than among women from the 2004 cohort study, however, no differences were found regarding ethnic origin, marital status, pre-pregnancy body mass index, antenatal risk, smoking during pregnancy and children who need special care after birth (Table 1). Follow-ups at twelve and 24 months after delivery were carried out in the whole cohort and the response rates were 94.3% and 93.5%, respectively. Missing information on the EPDS questionnaire was 0.8% and 1.2% in the twelve and 24-month follow-up, respectively.

**Table 1:** Comparison of maternal characteristics between women from the sample at the 3^rd ^month follow-up and women from the 2004 cohort study.

Variable	Sample %	Rest of the cohort %	p*
**Black/mixed ethnic origin**	28.0	27.0	0.540
**Maternal age**			0.448
≤ 19	19.0	18.9	
20–34	68.9	67.3	
>34	12.1	13.8	
**Single mother**	15.4	16.9	0.276
**Parity**			0.001
0	43.9	38.1	
1	26.6	25.9	
≥ 2	29.5	36.0	
**Pre-pregnancy body mass index (kg/m2)**			0.805
<18.5	5.5	4.8	
18.5 – 24.9	60.0	61.6	
25.0 – 29.9	23.4	23.0	
≥ 30	11.1	10.6	
**Antenatal risk**			0.231
Low	18.4	17.7	
Medium	69.1	67.5	
High	12.5	14.8	
**Smoked during pregnancy**	25.6	24.9	0.661
**Multiple birth**	2.3	1.0	0.018
**Had child with neonatal problems**	7.9	10.0	0.06

* chi-square test

**Figure 1:**
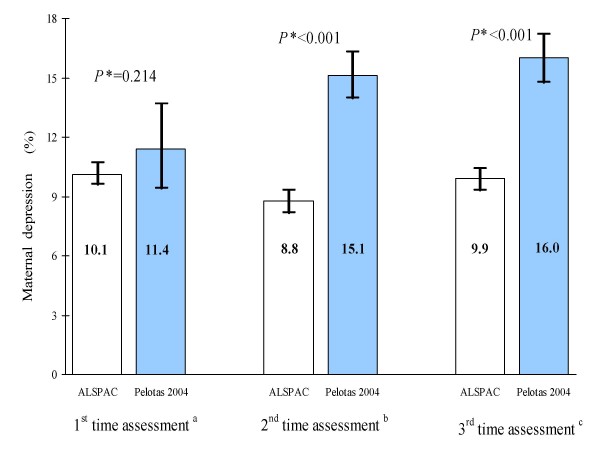
**Prevalence of maternal depression and 95% confidence intervals among ALSPAC and Pelotas birth cohort studies in the three time period studied**. ^a ^at 8 weeks and 3 months after delivery for ALSPAC and Pelotas birth cohort, respectively. ^b ^at 8 and 12 months after delivery for ALSPAC and Pelotas birth cohort, respectively. ^c ^at 21 and 24 months after delivery for ALSPAC and Pelotas birth cohort, respectively. * *x*^2 ^test for difference between the ALSPAC and the Pelotas birth cohort study in each time assessment

Variations in rates of maternal depression after childbirth between the ALSPAC and Pelotas birth cohort studies and across time-periods are shown in Figure 1. The rates of maternal depression were practically the same at the first time assessment for both studies. However, at the second and third time assessments, the rates observed in the Pelotas birth cohort study were consistently higher than in the ALSPAC study. In the ALSPAC study, rates of depression were almost the same in the three time assessments (*x*^2 ^trend p = 0.4), but in the Pelotas cohort study rates increased over time (*x*^2 ^trend p = 0.003).

Variations in rates of maternal depression after childbirth between the ALSPAC and Pelotas birth cohort studies and across time-periods are shown in Figure 1. The rates of maternal depression were practically the same at the first time assessment for both studies. However, at the second and third time assessments, the rates observed in the Pelotas birth cohort study were consistently higher than in the ALSPAC study. In the ALSPAC study, rates of depression were almost the same in the three time assessments (*x*^2 ^trend p = 0.4), but in the Pelotas cohort study rates increased over time (*x*^2 ^trend p = 0.003).

Marked differences in maternal characteristics were observed between the two cohort studies (Table 2). The Pelotas birth cohort study had higher frequencies of women of Black/mixed ethnic origin, with extremes ages (<20 and >34 year-old), single mothers and multiparae (≥ 2) than the ALSPAC study. Even though the proportion of pre-pregnancy underweight (BMI <18.5 kg/m^2^) was almost the same in both studies (5%), pre-pregnancy obesity (BMI > 30 kg/m^2^) was nearly two-times higher in the Pelotas than in the ALSPAC study. In addition, the proportion of women with high antenatal risk was higher in the Pelotas cohort as was the frequency of neonatal problems observed among their offspring. No differences were observed between the two studies regarding smoking during pregnancy and multiple birth.

**Table 2:** Comparison of maternal characteristics between ALSPAC (*n *= 13 798) and 2004 Pelotas (*n *= 4109) birth cohort studies.

Variable	ALSPAC *n *(%)	Pelotas *n *(%)	P *
**Ethnic origin**			<0.001
White	11855 (97.4)	3014 (73.4)	
Black/mixed	318 (2.6)	1095 (26.6)	
**Maternal age (y)**			<0.001
<20	649 (4.7)	773 (18.8)	
20–34	11780 (85.4)	2785 (67.8)	
≥ 35	1369 (9.9)	549 (13.4)	
**Marital status**			<0.001
With partner	11442 (97.5)	3451 (84.0)	
Single mother	288 (2.5)	658 (16.0)	
**Parity**			<0.001
0	5672 (45.1)	1624 (39.6)	
1	4388 (34.8)	1082 (26.3)	
≥ 2	2526 (20.1)	1402 (34.1)	
**Pre-pregnancy body mass index (kg/m^2^)**			<0.001
<18.5	570 (5.0)	187 (4.9)	
18.5 – 24.9	8472 (74.4)	2333 (61.3)	
25.0 – 29.9	1718 (15.1)	873 (23.0)	
≥ 30	627 (5.5)	409 (10.8)	
**Antenatal risk**			<0.001
Low	2678 (49.7)	717 (18.1)	
Medium	2268 (42.2)	2702 (68.0)	
High	435 (8.1)	551 (13.9)	
**Smoking during pregnancy**			0.565
No	8179 (75.9)	3099 (75.4)	
Yes	2601 (24.1)	1010 (24.6)	
**Multiple birth**			0.110
No	13617 (98.7)	4068 (99.0)	
Yes	181 (1.3)	41 (1.0)	
**Neonatal problems**			0.001
No	11261 (93.3)	3755 (91.7)	
Yes	813 (6.7)	342 (8.4)	

* chi-square

Variations in the rates of maternal depression across potential risk factors are shown in Table 3. At the three time assessments and in both studies, rates of maternal depression were highest among the poorest women, multiparae, those with high antenatal risk and women who smoked during pregnancy. In the ALSPAC study women of Black/mixed ethnic origin, adolescents, single mothers and women with pre-pregnancy underweight were more likely to be depressed at the three time assessments. In the Pelotas birth cohort study, women of Black/mixed ethnic origin and young mothers were more likely to be depressed in the 2^nd ^and 3^rd ^time assessments. Women with multiple birth were as likely to be depressed as women with singleton birth both in ALSPAC and Pelotas cohort study.

**Table 3:** Prevalence of maternal depression by time and association with potential determinants among ALSPAC (1991) and Pelotas (2004) birth cohort studies.

Variable	Postnatal depression **(time 1)**^a^		Postnatal depression **(time 2)**^b^		Postnatal depression **(time 3)**^c^	
	
	ALSPAC %	Pelotas %	ALSPAC %	Pelotas %	ALSPAC %	Pelotas %
**Ethnic origin**						
White	9.6	10.8	8.4	14.3	9.5	14.9
Black/mixed	18.4	13.0	13.7	17.2	18.4	19.1
*P *value*	<0.001	0.362	0.005	0.025	<0.001	0.002
**Family income (quintiles)**						
1^st^	15.2	15.5	12.0	22.1	15.6	22.7
2^nd^	9.9	17.2	8.2	20.0	11.1	19.7
3^rd^	7.0	8.2	8.3	14.5	8.8	16.1
4^th^	7.0	10.2	6.4	11.0	5.9	12.7
5^th^	6.1	6.3	6.0	8.1	6.7	8.9
*P *value*	<0.001	0.003	<0.001	<0.001	<0.001	<0.001
**Maternal age (y)**						
<20	18.6	15.1	13.9	18.4	14.7	17.0
20–34	9.6	11.3	8.3	14.3	9.6	15.8
≥ 35	11.4	6.5	10.7	14.7	10.7	15.6
*P *value*	<0.001	0.092	<0.001	0.024	0.008	0.733
**Marital status**						
With partner	9.5	10.0	8.3	14.6	9.3	15.6
Single mother	17.2	19.4	13.6	17.9	19.5	18.4
*P *value*	<0.001	0.002	0.004	0.033	<0.001	0.079
**Parity**						
0	8.6	8.3	6.7	11.9	8.3	12.7
1	10.2	10.6	9.8	12.9	9.9	12.4
≥ 2	12.4	16.9	10.1	20.5	12.5	22.6
*P *value*	<0.001	0.003	<0.001	<0.001	<0.001	<0.001
**Pre-pregnancy body mass index (kg/m^2^)**						
<18.5	14.4	20.0	12.1	19.8	15.3	14.7
18.5 – 24.9	9.4	10.3	8.2	14.9	9.0	15.7
25.0 – 29.9	9.6	7.7	8.2	13.4	10.3	14.6
≥ 30	12.1	15.1	10.4	14.5	10.2	16.6
*P *value*	0.001	0.050	0.011	0.186	<0.001	0.798
**Antenatal risk**						
Low	8.0	6.8	7.7	10.0	8.7	10.8
Medium	12.2	10.7	10.4	14.4	11.9	15.0
High	16.1	21.8	11.7	23.7	15.5	25.6
*P *value*	<0.001	<0.001	0.001	<0.001	<0.001	<0.001
**Smoking during pregnancy**						
No	8.0	7.6	7.1	12.8	8.4	13.5
Yes	15.4	22.6	12.2	22.3	13.4	23.7
*P *value*	<0.001	<0.001	<0.001	<0.001	<0.001	<0.001
**Multiple birth**						
No	10.1	11.6	8.7	15.0	9.8	16.0
Yes	11.6	0	13.3	20.5	11.8	18.0
*P *value*	0.565	0.279	0.058	0.341	0.481	0.738
**Neonatal problems**						
No	9.7	11.2	8.6	14.7	9.7	15.7
Yes	13.4	14.5	9.2	17.9	11.0	19.6
*P *value*	0.002	0.406	0.557	0.126	0.307	0.072

^a ^at 8 weeks and 3 months after delivery for ALSPAC and Pelotas birth cohort, respectively

^b ^at 8 and 12 months after delivery for ALSPAC and Pelotas birth cohort, respectively

^c ^at 21 and 24 months after delivery for ALSPAC and Pelotas birth cohort, respectively

* chi-square

Women from the Pelotas cohort study had a higher risk of having maternal depression after childbirth than women from the ALSPAC study in the unadjusted analyses (Table 4). After including socioeconomic and demographic factors and other maternal characteristics, the ORs were reduced. Because income quintiles were used in both cohorts, and by definition there will be equal proportions, this variable made no difference to the adjusted coefficients. However, had the distributions of all other maternal characteristics been the same between both cohort studies, women from the Pelotas cohort study would still present a higher risk of maternal depression at the second and third time assessments.

**Table 4:** Unadjusted and adjusted analyses for the association between maternal depression after childbirth and cohort study (ALSPAC cohort study = reference)

Maternal depression	Unadjusted OR (95% CI)	Adjusted OR * (95% CI)
Time 1 ^a^	1.15 (0.92; 1.42)	0.82 (0.64; 1.05)
Time 2 ^b^	1.85 (1.66; 2.07)	1.53 (1.34; 1.76)
Time 3 ^c^	1.74 (1.56; 1.94)	1.41 (1.23; 1.62)

^a ^at 8 weeks and 3 months after delivery for ALSPAC and Pelotas birth cohort, respectively

^b ^at 8 and 12 months after delivery for ALSPAC and Pelotas birth cohort, respectively

^c ^at 21 and 24 months after delivery for ALSPAC and Pelotas birth cohort, respectively

* adjusted for family income, race, age, marital status, parity, pre-pregnancy body mass index, antenatal risk, smoking during pregnancy, multiple birth and neonatal problems

OR = odds ratio; CI = confidence intervals

The results for income-related inequality in maternal depression after childbirth are shown in Table 5. In the unadjusted analysis, differences in the relative index of inequality (RII) between ALSPAC and Pelotas cohort study were observed mainly in the second time assessment, where the RII in Pelotas was higher than in the ALSPAC study. In the ALSPAC study, adjustment for covariates did not considerably alter any of the unadjusted RII. In the Pelotas cohort study, after adjustment for these factors there was a reduction in the magnitude of the RII at each time assessment. Overall, in the adjusted analyses no differences in income-related inequalities in maternal depression after childbirth were observed between the two studies at any time assessment.

**Table 5:** Relative index of inequality (RII) for maternal depression by time ranked by family income in the ALSPAC and the Pelotas birth cohort studies.

Time points	Unadjusted analysis		P*	Adjusted analysis**		P*
				
	ALSPAC RII (95% CI)	Pelotas RII (95% CI)		ALSPAC RII (95% CI)	Pelotas RII (95% CI)	
Time 1 ^a^	3.25 (2.50; 4.22)	3.17 (1.56; 6.42)	0.948	2.86 (1.90; 4.30)	1.58 (0.67; 3.74)	0.246
Time 2 ^b^	2.35 (1.79; 3.08)	3.51 (2.60; 4.74)	0.043	2.05 (1.34; 3.15)	2.33 (1.65; 3.31)	0.234
Time 3 ^c^	3.43 (2.65; 4.43)	3.01 (2.25; 4.02)	0.511	2.91 (1.93; 4.39)	2.25 (1.60; 3.16)	0.743

^a ^at 8 weeks and 3 months after delivery for ALSPAC and Pelotas birth cohort, respectively

^b ^at 8 and 12 months after delivery for ALSPAC and Pelotas birth cohort, respectively

^c ^at 21 and 24 months after delivery for ALSPAC and Pelotas birth cohort, respectively

* significance of the difference between ALSPAC and Pelotas birth cohort study

** adjusted for race, age, marital status, parity, pre-pregnancy body mass index, antenatal risk, smoking during pregnancy, multiple birth and neonatal problems.

## Discussion

Women from the Pelotas and ALSPAC cohorts showed similar risks of maternal depression at the first assessment point. However, at both the second and third time assessments, the likelihood of being depressed was higher in Pelotas. These differences were not completely explained by variations in maternal characteristics. Income-related inequalities in maternal depression after childbirth were high in both cohort studies at the three time assessments.

A major strength of the present study was the mode of data collection (prospective information obtained among large unselected populations and comparable timescales) combined with the use of a standardised and well validated screening instrument for maternal postnatal depression in both cohort studies. However, some methodological difficulties of the study need to be discussed. First, it is possible that rates of maternal depression among the ALSPAC study would be higher if no "attrition" was present. In ALSPAC, 8.1% of non-depressed mothers and 14.5% of depressed mothers at the first assessment failed to complete questionnaires at the second assessment point (*x*^2 ^p < 0.001) and 12.1% non-depressed and 17.1% depressed mothers at the second point have missing information at the third time assessment (*x*^2 ^p < 0.001). In the Pelotas cohort study there were no differences between depressed and non-depressed mothers in their likelihood of completing questionnaires at any time point. Secondly, although the proportion of missing values in family income, pre-pregnancy BMI and smoking during pregnancy from the ALSPAC database was relatively high, the use of multiple imputation analysis to assess the impact of missing values on the adjusted estimates provided some assurance against substantial selection bias [24]. In addition, all the analyses were repeated with no imputation for missing values: the risk estimates for the association between maternal depression after childbirth and cohort study, as well as the magnitude of the adjusted RII were very similar to those using the database with multiple imputation. Thirdly, we have no information about women who were treated with antidepressants at the time the EPDS was administered and some misclassification cannot be ruled out. Misclassification in this case would have erroneously classified depressed women as non-depressed. Medical treatment for depression depends upon maternal consultation with a health professional, recognition of the symptoms by the professional and maternal access to antidepressant medicines. Satisfaction of all these steps was more likely to happen among mothers from the ALSPAC cohort. Fourth, different timing of maternal depression assessments (8^th ^week *vs *3^rd ^month, 8^th ^*vs *12^th ^month and 21^st ^*vs *24^th ^month in ALSPAC and Pelotas cohort, respectively) could have lead to biases in the results. Especially for the 1^st ^and 2^nd ^assessments, differences in maternal depression after childbirth could have been smaller if the same timescale have been used in both studies. Finally, we are comparing data from two different time periods (ALSPAC with births in 1991–2 and the Pelotas cohort study with births in 2004). Several changes occurred among women in the UK in the last decade in areas such as employment, education and ethnic composition. Compared to women from Brazil, women from UK that feel depressed after childbirth have better access to treatment and more support from government and non-governmental organizations [25,26]. So, it is possible that the magnitude of the differences found in maternal depression between the two cohorts could be different and even larger, if data from closely matched time periods had been used.

At the first time assessment, the prevalence rate of maternal depression in the ALSPAC study was lower than the prevalence rates reported in other studies carried out in the UK. Using the EPDS and a cut-off at 13 points or above, Honey *et al *[27] found a prevalence of maternal depression at six weeks postpartum of 17%, similar to that reported by Thompson *et al *[28] of 19% at twelve weeks after delivery. However, the prevalence rate of maternal depression found in the Pelotas cohort study was very close to the prevalence rate of 12% reported by Da Silva *et al *[17] in the third month postpartum in Brazil. Studies carried out in other countries using the EPDS and a cut-off score of 13 or greater and similar time frame showed substantial variation in the prevalence rates of maternal depression. In Australia, Matthey *et al *[7] found a prevalence rate of 8% close to the prevalence rate of 9% found by Stamp et al [29], both studies at six weeks after delivery.

Using the EPDS with a cut-point of ≥ 13, Luoma *et al *[30], in Finland, reported a prevalence of maternal depression at two months after giving birth of 9% and Righetti-Veltema *et al *[31], in Switzerland, using the same test and cut-point reported a prevalence of 10.2% at three months after delivery. In Denmark, Nielsen Forman *et al *[32], reported a prevalence of 5.5% at four months after childbirth. However, in Turkey, Bugdayci *et al *[33], using the EPDS with a cut-point of ≥ 13 reported a prevalence rate of 29% within zero and two months and 37% within the third and sixth months after delivery.

At the second and third time assessments the prevalence rates of maternal depression among women from the Pelotas cohort study were higher than those observed in the ALSPAC study. Studies carried out in other countries showed again great variability. Using EPDS with a cut-point of ≥ 13, Bernazzani *et al *[34] reported a prevalence of maternal depression at six-seven months postpartum of 12.7% in Canada while in Australia, Brown & Lumley [35] reported a prevalence of 16.9%. However, in a study carried out in Turkey, Bugdayci *et al *[33] reported a prevalence rate of maternal depression of 36% within seven and twelve months after delivery and 43% after thirteen months postpartum.

Even though there is great variability in the reported occurrence of postpartum depressive symptomatology between and within countries [9], lower levels of maternal depression after childbirth seemed to be found in Australia and western European countries, where postnatal depression has long being recognized as a health problem and treatment programs have been designed for their populations [8].

There is no consensus about the course of maternal depression during the first year postpartum. While some investigations reported remission in diagnosed depression over the course of the first postpartum year [36], other authors reported an increase. Rubertsson *et al *[37] in a national Swedish sample reported a prevalence of women with high EPDS scores (≥ 12) of 11.1% at two months and 13.7% one year after giving birth. In the ALSPAC study, rates of maternal depression remained almost stable over the first two years postpartum, while in the Pelotas cohort study, they increased over time. Despite the difference in maternal depression prevalence between the two populations, these results pointed at further need to investigate care-seeking behaviour for mental health professionals and availability of drug treatment for women in each setting. Lack or inadequate treatment of maternal depression after childbirth can explain its persistence throughout childbearing years with detrimental effects for mothers, their off-spring and the whole family.

The Pelotas cohort study had higher frequencies of Black/mixed ethnic origin women, with more extremes ages, single mothers and multiparae than the ALSPAC study. Several investigations have identified these characteristics as predictors of postpartum depression since they have direct and indirect relationships to postnatal depressive symptomatology [5,6,31,34]. In agreement with several authors, women living in disadvantaged economical conditions were at higher risk of depressive symptoms during the childbearing years [5,6]. Higher prevalence of maternal depression was found among Black/mixed women in our study, a finding that has been reported in both developed [38] and developing countries [17]. However, some authors pointed out that the higher prevalence of postpartum depressive symptoms seen among minority mothers could be entirely explained by financial hardship [5]. Our finding that multiparae had higher frequencies of depression is consistent with previous studies that reported a strong association between parity and vulnerability to postnatal depression [39]. However, other investigators failed to find an association in the adjusted analysis [37]. Both in the ALSPAC and in the Pelotas cohort study, high antenatal risk was associated with greater prevalence of maternal depression. The risk score used to evaluate antenatal risk took into account previous unfavorable obstetric outcomes (previous abortion, stillbirth, infant death) as well as maternal sociodemographic characteristics. Earlier investigations failed to find an association between either obstetric or perinatal complications of the mothers and their offspring and postnatal depression [40]. These results emphasize the importance of psychosocial risk factors for maternal depression.

Women from the Pelotas cohort study had higher prevalence of risk factors known to be associated with postnatal depression than women from the ALSPAC study. Nevertheless, the higher risk of maternal depression persisted in Pelotas at the second and third time assessments even after adjustment for other risk factors. It is likely that women from the Pelotas cohort study would suffer greater exposure to adverse conditions, not taken into account in our study, such as stressful life events and lower social and partner support, increasing the risk for maternal depression after childbirth in this population.

Although the burden of maternal depression was higher in the Pelotas cohort study, income-related inequalities were high in both cohorts. In the crude analyses, inequality was greater in Pelotas at 8–12 months, but not on the other two assessments. After adjustment for covariates – some of which may mediate the effect of income on depression, inequalities had similar magnitude in both the ALSPAC and the Pelotas birth cohort study. Mangalore *et al *[41] using data from the Psychiatric Morbidity Survey of 2000 for Britain found significant income-related inequality for psychiatric disorders, highlighting that these inequalities were higher than income-related inequalities for general health in the UK.

## Conclusion

Significant differences in maternal depression after childbirth between populations from Brazil and UK were observed in this study. At the second and third time assessments the prevalence rates of maternal depression among women from the Pelotas cohort study were higher than those observed in the ALSPAC study even after adjustment for potentially confounding factors. The results of this study strengthen the contention that the burden of maternal depression after childbirth varies between and within populations. However, strategies to reduce income-related inequalities in maternal depression should be targeted to low-income women in both developed and developing countries.

## Competing interests

The authors declare that they have no competing interests.

## Authors' contributions

AM and JG conceived the research question. AM conducted the analyses and wrote the first draft of the study. GDS and JG supervised the analysis and interpretation of the findings as well as the writing of the paper. CGV, ISS and AJDB contributed to the interpretation of the analyses and assisted with the editing of the article. All authors approved the final version of the manuscript.
